# Supplementary material for: A glutamatergic DRN–VTA pathway modulates neuropathic pain and comorbid anhedonia-like behavior in mice
Source: Nat Commun. 2023 Aug 23;14:5124. doi: 10.1038/s41467-023-40860-3 (PMC10447530; doi:10.1038/s41467-023-40860-3)
Supplement: Supplementary file 1 — Supplementary Information [file 41467_2023_40860_MOESM1_ESM.pdf]

# A glutamatergic DRN–VTA pathway modulates neuropathic pain and comorbid anhedonia-like behavior in mice

Xin-Yue Wang[1*], Wen-Bin Jia[1*], Xiang Xu[1], Rui Chen[1], Liang-Biao Wang[1], Xiao-Jing Su[1], Peng-Fei Xu[1], Xiao-Qing Liu[2], Jie Wen[3], Xiao-Yuan Song[4], Yuan-Yuan Liu[5], Zhi Zhang[4†], Xin-Feng Liu[1†] and Yan Zhang[1†]

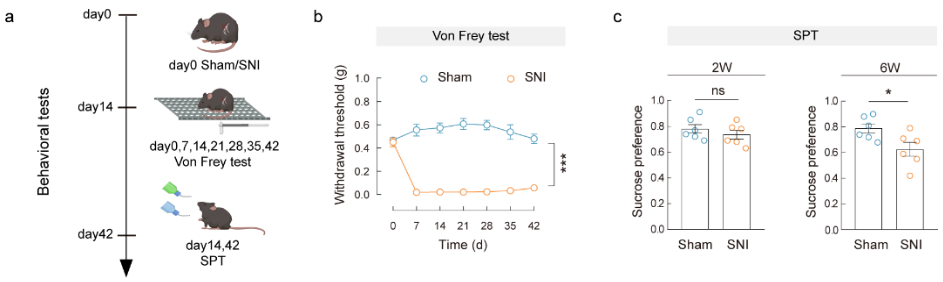


**Supplementary Fig 1. Time course of SNI-induced mechanical hypersensitivity and comorbid anhedonia-like behavior.**

**a**, Schematic of the experimental design. **b**, **c**, Performance of mice treated with sham or SNI in von Frey test (**b**: Sham, n = 6; SNI, n = 5 mice. $P = 0.0009$) and SPT (**c**: Sham, n = 6; SNI, n = 6 mice. 2W $P = 0.3892$; 6W $P = 0.0312$). Significance was assessed by two-way ANOVA followed by Bonferroni's multiple comparisons test in (**b**), and two-tailed unpaired Student's *t*-test in (**c**). All data are presented as the mean ± s.e.m. $^{*}P < 0.05$, $^{***}P < 0.001$, not significant (ns). Details of the statistical analyses are presented in Supplementary Data 1. Created with BioRender.com (**a**).

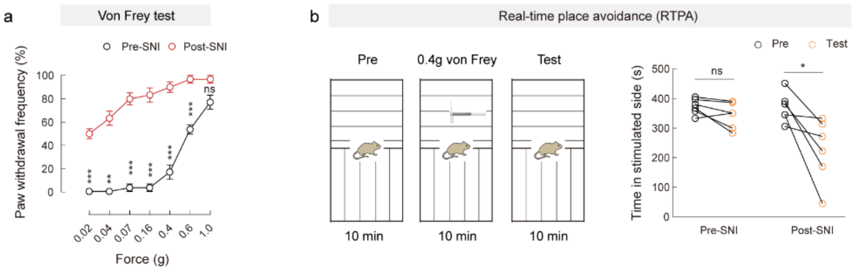


**Supplementary Fig 2. Mechanical withdrawal frequency to von Frey filament and RTPA test before and after SNI.**

**a**, Statistical data for mechanical withdrawal frequencies to von Frey filaments applied on the hind paw in *C57* mice before (pre-SNI) and after (post-SNI 2W) SNI. **b**, Experimental design of real-time place avoidance (RTPA) test (left) and quantification of RTPA before (Pre) and after (Test) training of the pre-SNI ($P = 0.0995$) or post-SNI 2W ($P = 0.0237$) mice (right). n = 6 mice per group in panels (**a**, **b**). Significance was assessed by two-way ANOVA followed by Bonferroni's multiple comparisons test in (**a**) and two-tailed paired Student's *t*-test in (**b**). All data are presented as the mean ± s.e.m. $*P < 0.05$, $**P < 0.01$, $***P < 0.001$, not significant (ns). Details of the statistical analyses are presented in Supplementary Data 1.

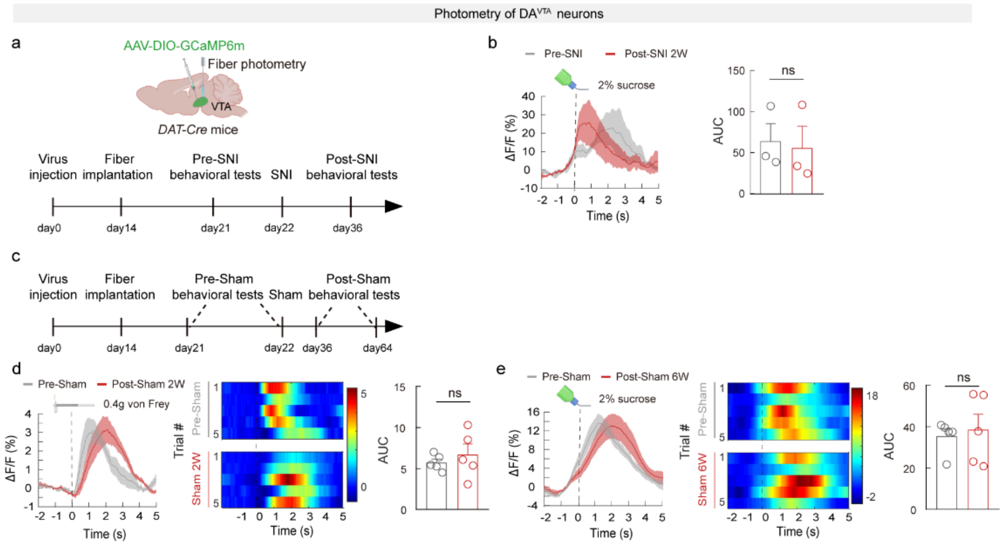


**Supplementary Fig 3. Ca$^{2+}$ signal of DA$^{VTA}$ neurons in *DAT-Cre* mice.**

**a**, Schematic of the experimental design and schematic diagram of fiber photometry of DA$^{VTA}$ neurons. **b**, Averaged responses (left) and AUC during 0-5 s (right) showing Ca$^{2+}$ responses evoked by sucrose licking in pre- and post-SNI 2W mice. (n = 3 mice. *P* = 0.2400). **c**, Schematic of the experimental design. **d**, **e**, Averaged responses (left), heatmaps (middle), and AUC during 0-5 s (right) showing Ca$^{2+}$ responses evoked by 0.4g von Frey stimulation in pre- and post-Sham 2W mice (**d**: n = 5 mice. *P* = 0.4480) and sucrose licking in pre- and post-Sham 6W mice (**e**: n = 5 mice. *P* = 0.6404). Significance was assessed by two-tailed paired Student's *t*-test in (**b**, **d**, **e**). All data are presented as the mean ± s.e.m. Not significant (ns). Details of the statistical analyses are presented in Supplementary Data 1. Created with BioRender.com (**a**, **b**, **d**, **e**).

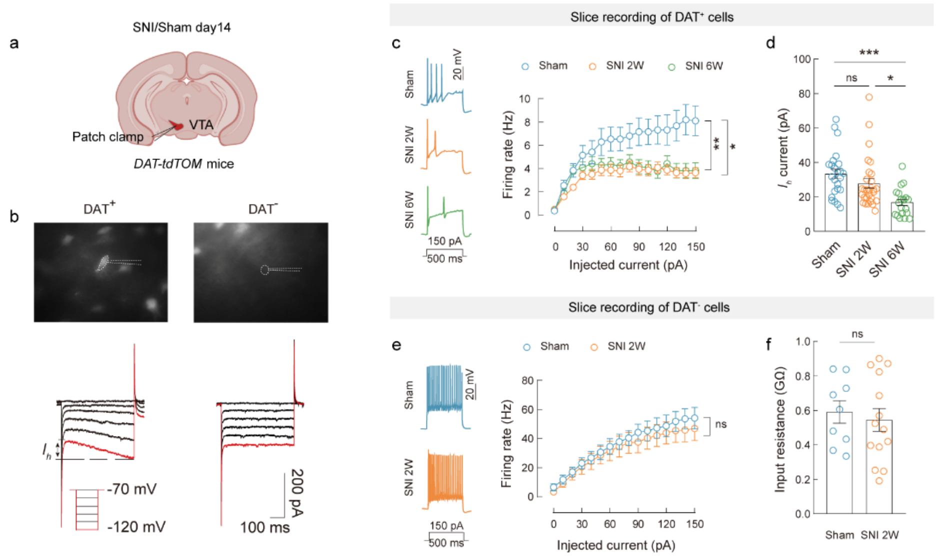


**Supplementary Fig 4. The excitability of VTA neurons in SNI and Sham mice.**

**a**, Schematic of the electrophysiological recordings in acute slices. **b**, Typical currents induced by hyperpolarizing voltage steps recorded from the DAT$^{+}$ (tdTOM positive) and DAT$^{-}$ (tdTOM negative) neurons. **c**, Representative traces (left) and statistical data (right) of firing rate recorded from DAT$^{+}$ neurons of Sham, post-SNI 2W, and post-SNI 6W mice. Sham, n = 23 cells from 3 mice; SNI 2W, n = 27 cells from 3 mice; SNI 6W, n = 30 cells from 4 mice. Sham vs SNI 2W $P = 0.0056$; Sham vs SNI 6W $P = 0.0112$. **d**, Statistical data of hyperpolarization-activated currents ($I_h$) at -120 mV recorded from DAT$^{+}$ neurons. Sham, n = 24 cells from 3 mice; SNI 2W, n = 28 cells from 3 mice; SNI 6W, n = 20 cells from 3 mice. Sham vs SNI 2W $P = 0.3945$; Sham vs SNI 6W $P = 0.0002$; SNI 2W vs SNI 6W $P = 0.0151$. **e**, Representative traces and statistical data of firing rate recorded from DAT$^{-}$ neurons of post-SNI 2W and Sham mice. Sham, n = 8 cells from 3 mice; SNI 2W, n = 11 cells from 3 mice. $P > 0.9999$. **f**, Statistical data for input resistance recorded from DAT$^{-}$ neurons of post-SNI 2W and Sham mice. Sham, n = 9 cells from 3 mice; SNI 2W, n = 14 cells from 3 mice. $P = 0.6418$. Significance was assessed by two-way ANOVA followed by Bonferroni's multiple comparisons test in (**c, e**), one-way ANOVA followed by Bonferroni's multiple comparisons test in (**d**), and two-tailed unpaired Student's *t*-test in (**f**). All data are presented as the mean ± s.e.m. $^{*}P < 0.05$, $^{**}P < 0.01$, $^{***}P < 0.001$, not significant

(ns). Details of the statistical analyses are presented in Supplementary Data 1. Created with BioRender.com (**a**).

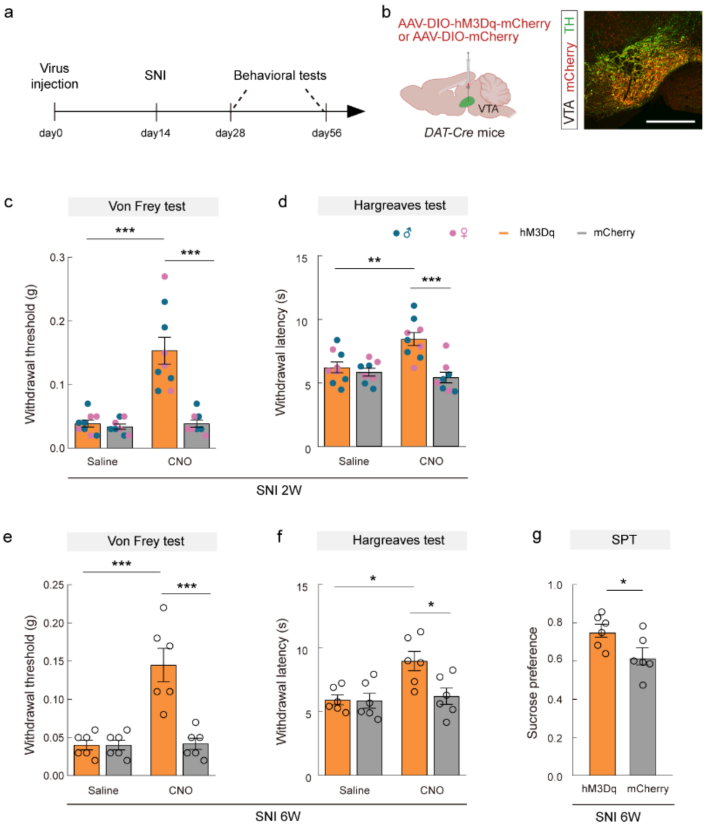


**Supplementary Fig 5. Effects of chemogenetic activation of $DA^{VTA}$ neurons on chronic pain.**

**a**, Schematic of the experimental design. **b**, Schematic of VTA injection of AAV-DIO-hM3Dq-mCherry/AAV-DIO-mCherry (left) in *DAT*-Cre mice and representative images of mCherry-expressing neurons co-localized with TH immunofluorescence in the VTA (right). Scale bar, 500 μm. **c-f**, Statistical data for mechanical paw withdrawal threshold (**c**: hM3Dq, n = 9; mCherry, n = 8. $P < 0.0001$**,** **e**: hM3Dq, n = 6; mCherry, n = 6. $P < 0.0001$), thermal paw withdrawal latency (**d**: hM3Dq, n = 9; mCherry, n = 8. hM3Dq&CNO vs hM3Dq&Saline $P = 0.0045$; hM3Dq&CNO vs mCherry&CNO $P = 0.0002$, **f**: hM3Dq, n = 6; mCherry, n = 6. hM3Dq&CNO vs hM3Dq&Saline $P = 0.0115$; hM3Dq&CNO vs mCherry&CNO $P = 0.0248$), and SPT (**g**: hM3Dq, n = 6; mCherry, n = 6. $P = 0.0374$) of hM3Dq-mCherry-expressing and mCherry-expressing SNI mice with saline or CNO treatment. Significance was assessed by two-way ANOVA followed by Bonferroni's

multiple comparisons test in (**c**-**f**) and two-tailed unpaired Student's *t*-test in (**g**). All data are presented as the mean ± s.e.m. *$P < 0.05$, **$P < 0.01$, ***$P < 0.001$. Details of the statistical analyses are presented in Supplementary Data 1. Created with BioRender.com (**b**).

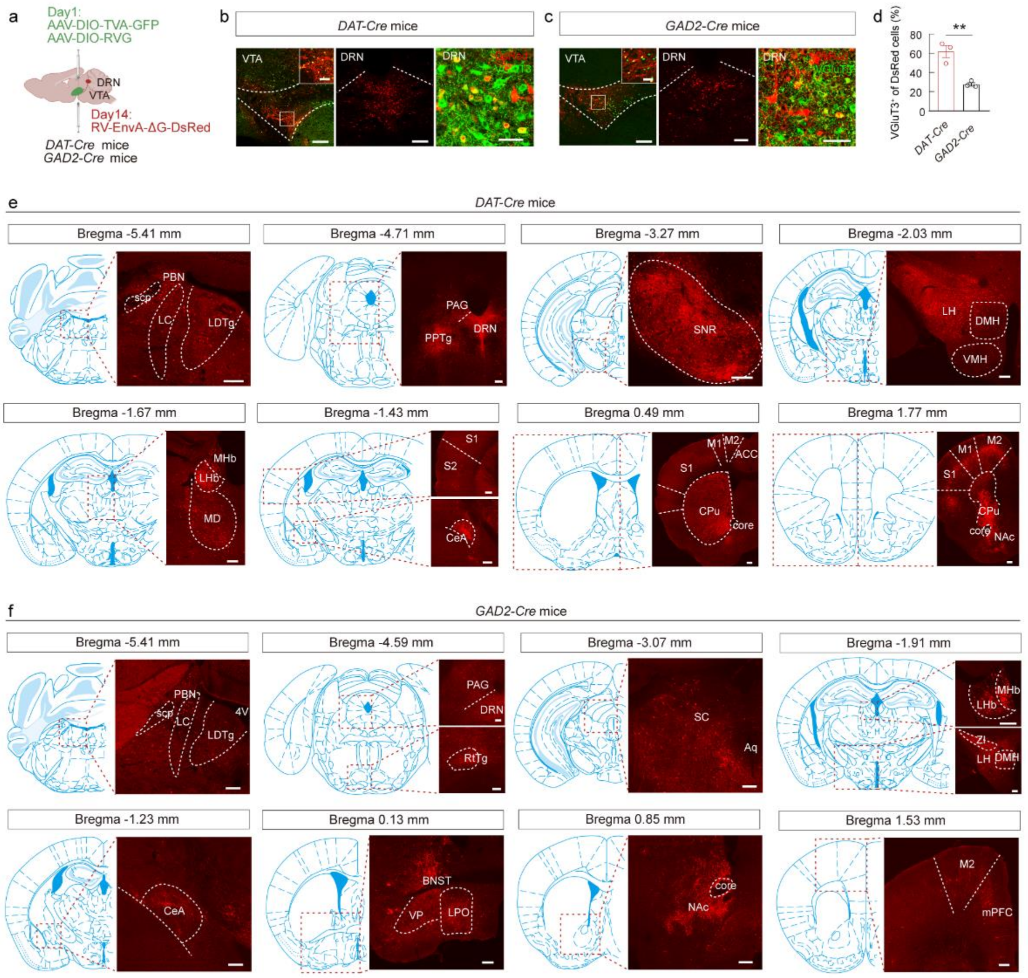


**Supplementary Fig 6. Mapping of monosynaptic inputs to $DA^{VTA}$ and $GABA^{VTA}$ neurons.**

**a**, Schematic of the Cre-dependent retrograde monosynaptic tracing strategy in *DAT-Cre* and *GAD2-Cre* mice. **b**, **c**, Representative images of the starter cells in the VTA (left) and RV-DsRed-labeled cells in the DRN (middle) which co-localize with VGluT3 immunofluorescence (right) from *DAT-Cre* (**b**) mice and *GAD2-Cre* mice (**c**). Starter cells (yellow) co-express AAV-DIO-TVA-GFP, AAV-DIO-RVG (green), and rabies RV-EnvA-ΔG-DsRed (red). Scale bars, 200 μm (left, middle) and 50 μm (upper, right). **d**, Summary data for the percentage of DsRed-labeled neurons that expressed VGluT3 in *DAT-Cre* and *GAD2-Cre* mice, n = 9 sections from 3 mice. $P = 0.0068$. **e**, **f**, Sample images showing DsRed-expressing cells (red) that make monosynaptic inputs onto $DA^{VTA}$ neurons or $GABA^{VTA}$ neurons. Scale bars, 200 μm. PBN, parabrachial

nucleus; LC, locus coeruleus; LDTg, laterodorsal tegmentum; PAG, periaqueductal gray; DRN, dorsal raphe nucleus; PPTg, pedunculopontine tegmentum; SNR, substantia nigra pars reticulata; LH, lateral hypothalamus; DMH, dorsomedial hypothalamus; VMH, ventromedial hypothalamus; LHb, lateral habenular nucleus; MHb, medial habenular nucleus; MD, mediodorsal thalamic nucleus; CeA, central nucleus of the amygdala; CPu, caudate putamen; S1, primary somatosensory cortex; S2, secondary somatosensory cortex; M1, primary motor cortex; M2, secondary motor cortex. ACC, anterior cingulate cortex; NAc, nucleus accumbens; BNST, bed nucleus of the stria terminalis; RtTg, reticulotegmental nucleus of the pons; ZI, zona incerta; SC, superior colliculus; VP, ventral pallidum; LPO, lateral preoptic area; mPFC, medial prefrontal cortex. Significance was assessed by two-tailed unpaired Student's *t*-test in (**d**). Data in (**d**) are presented as the mean ± s.e.m. $^{**}P < 0.01$. Details of the statistical analyses are presented in Supplementary Data 1. Created with BioRender.com (**a**).

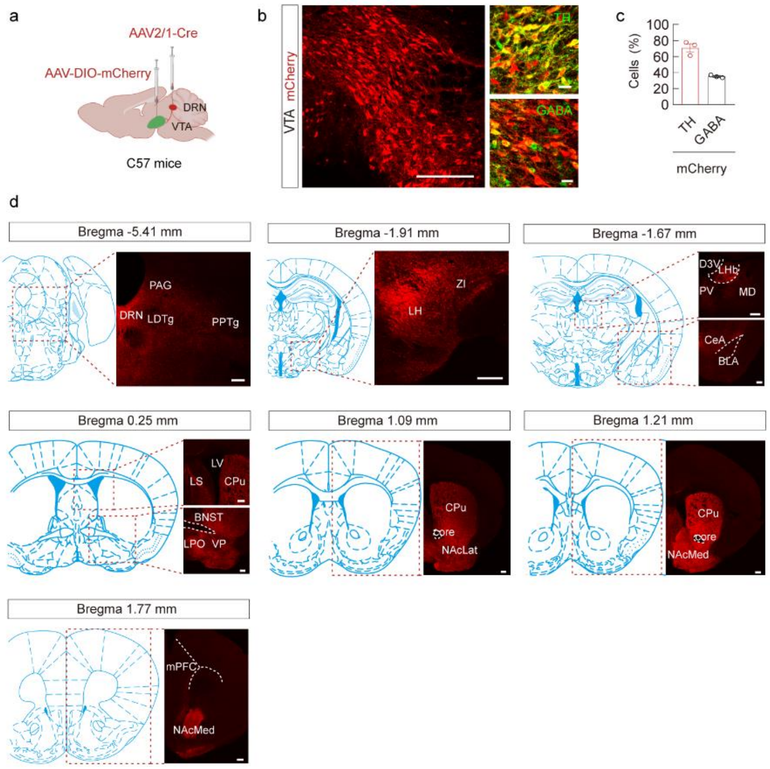


**Supplementary Fig 7. Cre-dependent anterograde trans-monosynaptic tracing of DRN neurons.**

**a**, Schematic of the Cre-dependent anterograde trans-monosynaptic tracing strategy in *C57* mice. **b**, Typical images showing mCherry expression within the VTA. Scale bars, 200 μm (left) and 100 μm (right). **c**, Summary data for the percentage of mCherry-labeled neurons co-localized with TH and GABA immunofluorescence within the VTA, n = 6 sections from 3 mice (right). **d**, Neural terminals expressing mCherry in different brain regions. Scale bars, 200 μm. PBN, parabrachial nucleus; LDTg, laterodorsal tegmentum; PAG, periaqueductal gray; DRN, dorsal raphe nucleus; PPTg, pedunculopontine tegmentum; LH, lateral hypothalamus; ZI, zona incerta; LHb, lateral habenular nucleus; PV, paraventricular thalamic nucleus; MD, mediodorsal thalamic nucleus; BLA, basolateral amygdala; CeA, central nucleus of the amygdala; LS, lateral septal nucleus; CPu, caudate putamen; LPO, lateral preoptic area; BNST, bed nucleus of the stria terminalis; VP, ventral pallidum; NAcLat, NAc lateral shell; NAcMed, NAc medial shell; mPFC, medial prefrontal cortex. Data in (**c**) are

presented as the mean ± s.e.m. Created with BioRender.com (**a**).

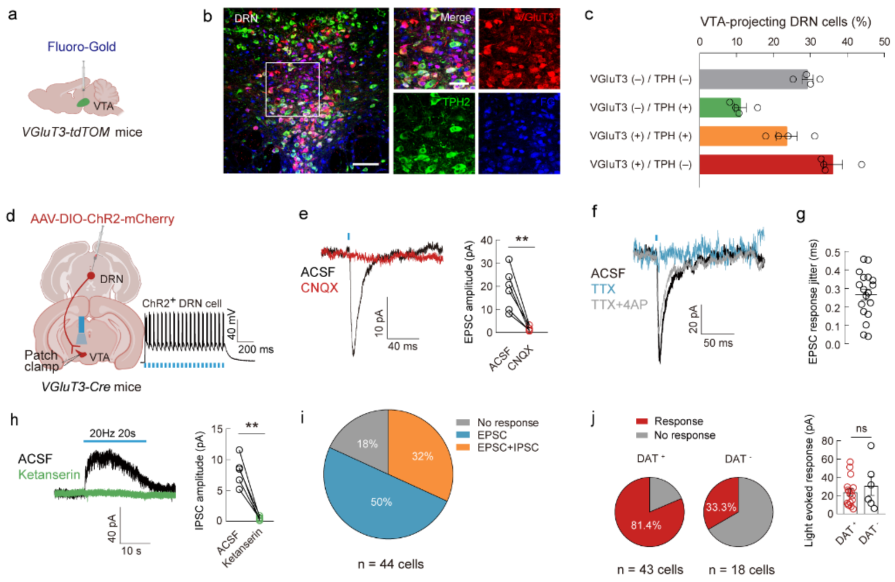


**Supplementary Fig 8. Dopamine neurons are the primary postsynaptic target of the VGluT3$^{DRN}$ neurons.**

**a**, Schematic diagram of retrograde tracer FG injected into the VTA. **b**, Typical images of the FG (blue) neurons co-localize with TPH2 immunofluorescence (green) in the DRN of *VGluT3-tdTOM* mice. Scale bars, 100 μm (left) and 50 μm (right). **c**, Percentage of FG-labeled neurons that expressed VGluT3 and TPH2 in the DRN, n = 15 sections from four mice. **d**, Schematic showing viral injection and VTA electrophysiological recordings in acute slices from *VGluT3-Cre* mice (left). Viral expression efficacy proved by light (473 nm, 20 Hz)-evoked action potentials of ChR2-mCherry-expressing neuron (right). **e**, Representative traces and summary data for light-evoked EPSCs of VTA neurons before (ACSF) and after CNQX (10 μM) treatment. n = 6 cells from 3 mice. *P* = 0.0040. **f**, Representative traces of light-evoked EPSCs of the VTA neurons before (ACSF) and after TTX (1 μM) or TTX and 4-AP (100 μM) treatment. **g**, Summary data for the jitter of light-evoked EPSCs recorded from VTA neurons. n = 18 cells from 3 mice. **h**, Representative traces and summary data for light-evoked (473 nm, 20 s, 20 Hz) slow IPSCs of VTA neurons before and after ketanserin (10 μM) treatment. n = 5 cells from 3 mice. *P* = 0.0019. **i**, Pie chart showing the distribution of light-evoked response types in

DRN-targeted VTA neurons. n = 44 cells from 5 mice. **j**, Summary data for the percentage (left: DAT$^{+}$, n = 43 cells from 5 mice; DAT$^{-}$, n = 18 cells from 3 mice) and amplitudes (right: DAT$^{+}$, n = 17 cells from 4 mice; DAT$^{-}$, n = 6 cells from 3 mice. $P$ = 0.4247) of light-evoked fast EPSCs recorded from DAT$^{+}$ and DAT$^{-}$ neurons. Significance was assessed by two-tailed paired Student's $t$-test in (**e**, **h)**, and two-tailed unpaired Student's $t$-test in (**j**). All data are presented as the mean ± s.e.m. **$P$ < 0.01, not significant (ns). Details of the statistical analyses are presented in Supplementary Data 1. Created with BioRender.com (**a**, **d**).

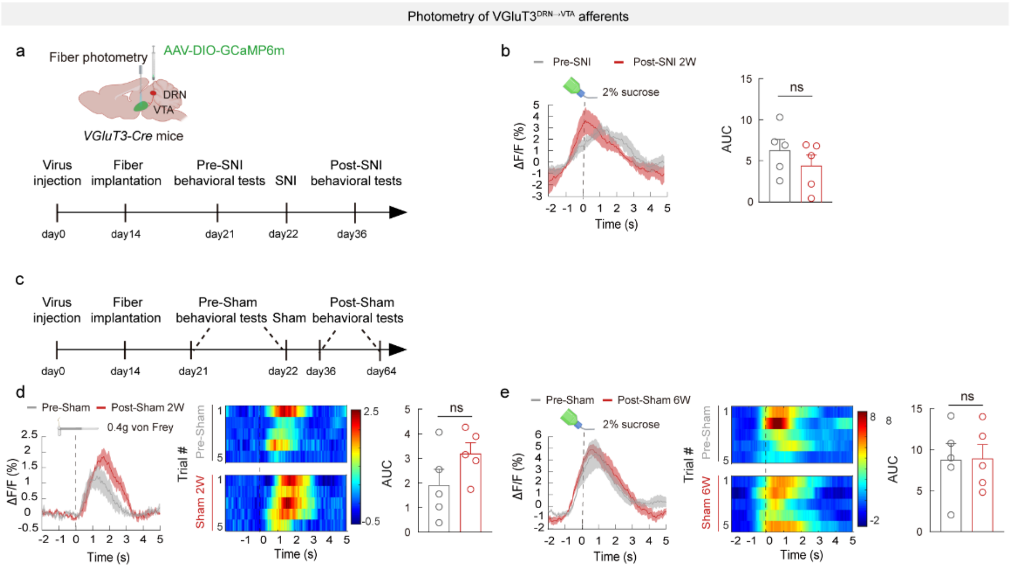


**Supplementary Fig 9. $Ca^{2+}$ signal of $VGluT3^{DRN\rightarrow VTA}$ afferents.**

**a**, Schematic of the experimental design and schematic diagram of fiber photometry of *VGluT3-Cre* mice. **b**, Averaged responses (left) and AUC during 0-5 s (right) showing $Ca^{2+}$ responses evoked by sucrose licking in pre- and post-SNI 2W mic. $P = 0.2607$. **c**, Schematic of the experimental design. **d**, **e**, Averaged responses (left), heatmaps (middle), and AUC during 0-5 s (right) showing $Ca^{2+}$ responses evoked by 0.4g von Frey stimulation in pre- and post-Sham 2W mice (**d**: $P = 0.1247$) and sucrose licking in pre- and post-Sham 6W mice (**e**: $P = 0.9502$). n = 5 mice per group in panels (**b**, **d**, **e**).Significance was assessed by two-tailed unpaired Student's *t*-test in (**b**, **d**, **e**). All data are presented as the mean ± s.e.m. Not significant (ns). Details of the statistical analyses are presented in Supplementary Data 1. Created with BioRender.com (**a**, **b**, **d**, **e**).

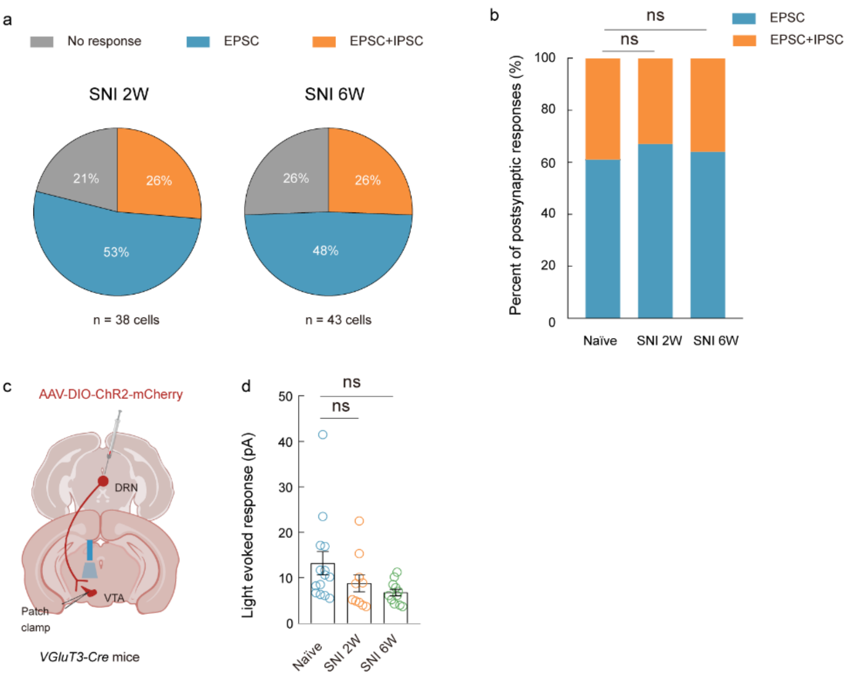


**Supplementary Fig 10. Analysis of nerve injury-induced changes in the postsynaptic responses of VGluT3$^{\mathrm{DRN}}$-targeted VTA neurons.**

**a**, Pie chart showing the distribution of light-evoked response types in VGluT3$^{\mathrm{DRN}}$-targeted VTA neurons of post-SNI 2W and post-SNI 6W mice. naïve vs SNI 2W *P* = 0.8502; naïve vs SNI 6W *P* = 0.6552. **b**, A bar graph illustrating the percentage of EPSC-type and EPSC+IPSC-type responses in all postsynaptic responses recorded from naïve (n = 44 cells from 5 mice), post-SNI 2W (n = 38 cells from 4 mice) and 6W mice (n = 43 cells from 4 mice). naïve vs SNI 2W *P* = 0.4615; naïve vs SNI 6W *P* = 0.7703. **c**, Schematic showing viral injection and the electrophysiological recordings in acute slices from *VGluT3-Cre* mice. **d,** Statistical data for the amplitudes of light-evoked slow IPSCs in VGluT3$^{\mathrm{DRN}}$-targeted VTA neurons. (naïve, n = 14 cells from 3 mice; post-SNI 2W, n = 10 cells from 3 mice; post-SNI 6W, n = 11 cells from 3 mice. naïve vs SNI 2W *P* = 0.2866; naïve vs SNI 6W *P* = 0.0609). Significance was assessed by two-tailed Chi-square test between groups in (**a**), two-tailed Fisher's exact test in (**b**), and one-way ANOVA followed by Bonferroni's multiple comparisons test in (**d**). All data are presented as the mean ±

s.e.m. Not significant (ns). Details of the statistical analyses are presented in Supplementary Data 1. Created with BioRender.com (**c**).

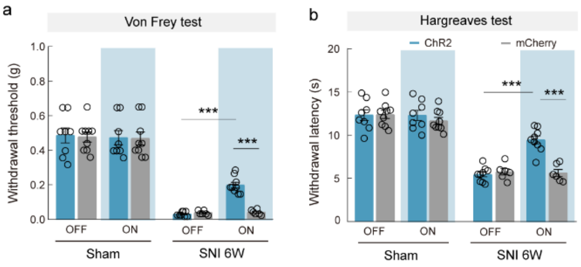


**Supplementary Fig 11. $VGluT3^{DRN}\rightarrow DA^{VTA}$ circuit contributes to analgesic effects in post-SNI 6W mice.**

**a**, **b**, Mechanical paw withdrawal threshold (**a**: ChR2&ON vs ChR2&OFF $P < 0.0001$; ChR2&ON vs mCherry&ON $P < 0.0001$) and thermal paw withdrawal latency (**b**: ChR2&ON vs ChR2&OFF $P < 0.0001$, ChR2&ON vs mCherry&ON $P < 0.0001$) of ChR2-mCherry-expressing and mCherry-expressing Sham or post-SNI 6W mice with (on) or without (off) optogenetic stimulation. Sham&ChR2, n = 8; Sham&mCherry, n = 9; SNI&ChR2, n = 9; SNI&mCherry, n = 6. Significance was assessed by two-way ANOVA followed by Bonferroni's multiple comparisons test in (**a**, **b**). All data are presented as the mean ± s.e.m. ***$P < 0.001$, not significant (ns). Details of the statistical analyses are presented in Supplementary Data 1.

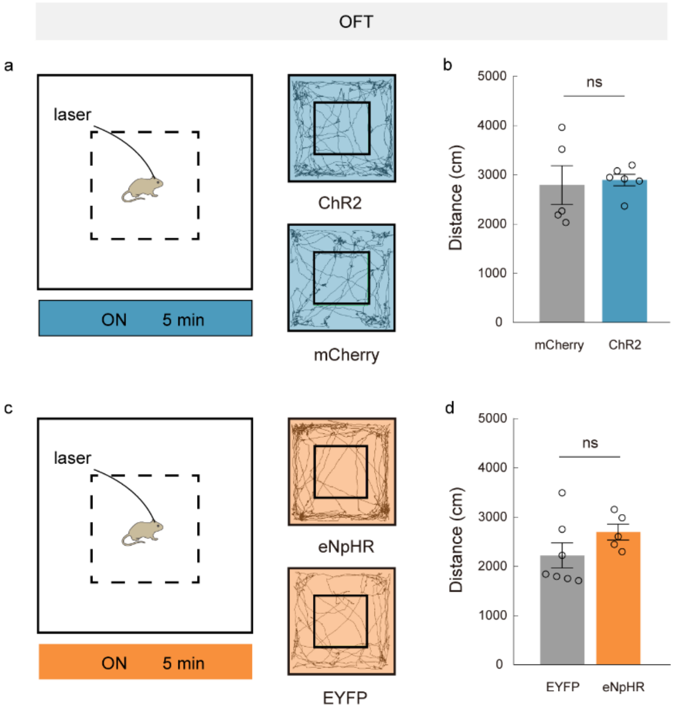


**Supplementary Fig 12. Motor activity in mice with light stimulation of VGluT3$^{DRN\rightarrow VTA}$ afferents.**

**a**, **c** Experimental design (left) and representative trajectories (right) of animals expressing ChR2/mCherry (**a**) or eNpHR/EYFP (**c**) in VGluT3$^{DRN}$ neurons during VTA light stimulation. **b**, **d**, Mean distance of ChR2/mCherry-expressing (**b**: ChR2, n = 6; mCherry, n = 5. *P* = 0.7904) and eNpHR/EYFP-expressing (**d**: eNpHR, n = 5; EYFP, n = 7. *P* = 0.1855) mice with 20 Hz optostimulationin OFT. Significance was assessed by two-tailed unpaired Student's *t*-test in (**b**, **d**). All data are presented as the mean ± s.e.m. Not significant (ns). Details of the statistical analyses are presented in Supplementary Data 1.

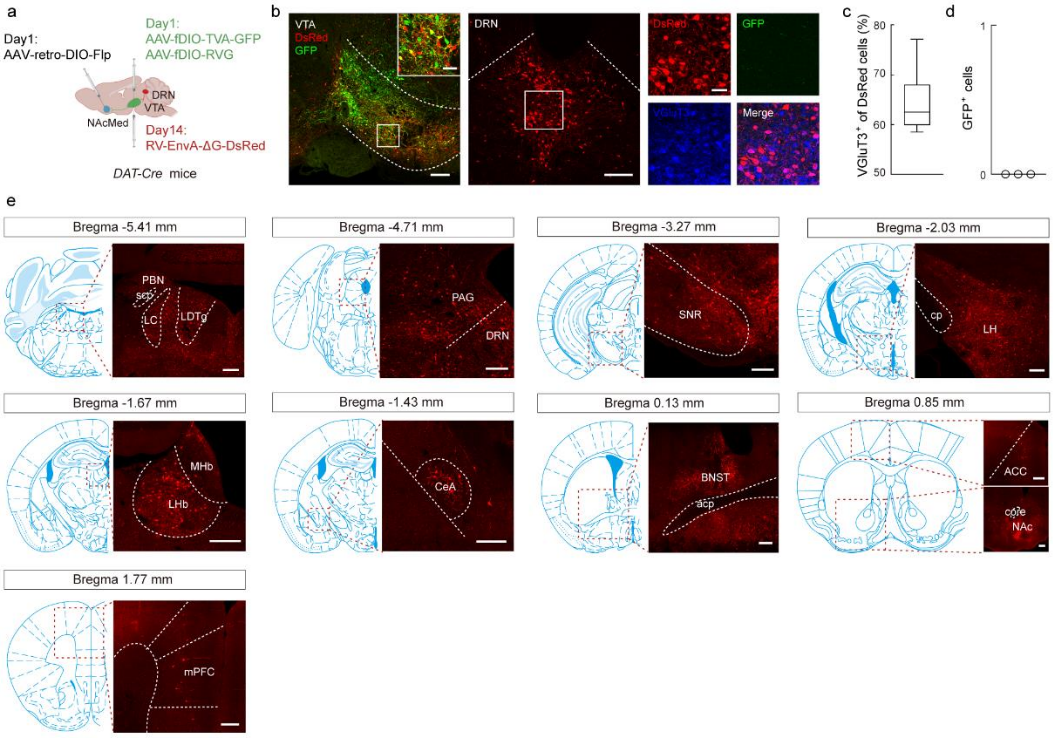


**Supplementary Fig 13. Presynaptic input to NAcMed-projecting DA$^{VTA}$ neurons revealed by cTRIO-based mediated trans-synaptic tracing.**

**a**, Schematic of cTRIO based retrograde monosynaptic tracing using *DAT-Cre* mice. **b**, Representative images of the starter cells in the VTA (left) and RV-DsRed-labeled cells in the DRN (middle) which co-localize with VGluT3 immunofluorescence (right). Starter cells (yellow) co-expressing AAV-fDIO-TVA-GFP, AAV-fDIO-RVG (green), and rabies RV-EnvA-ΔG-DsRed (red). Scale bars, 50 µm (upper) and 200 µm (bottom). **c**, Percentage of DsRed-labeled neurons in the DRN that express VGluT3 in *DAT-Cre* mice, n = 9 sections from three mice. **d**, GFP-expressing neurons in the DRN. n = 3 mice. **e**, Representative images showing DsRed-expressing cells (red) that make monosynaptic contact onto NAcMed-projecting DA$^{VTA}$ neurons. Scale bars, 200 µm. PBN, parabrachial nucleus; LC, locus coeruleus; LDTg, laterodorsal tegmentum; PAG, periaqueductal gray; DRN, dorsal raphe nucleus; SNR, substantia nigra pars reticulata; LH, lateral hypothalamus; LHb, lateral habenular nucleus; MHb, medial habenular nucleus; CeA, central nucleus of the amygdala; BNST, bed nucleus of the stria terminalis; ACC, anterior cingulate cortex; NAc, nucleus accumbens; mPFC,

medial prefrontal cortex. Data in (**c**) are shown as box and whisker plots (medians, quartiles (boxes), and ranges minimum to maximum (whiskers). Created with BioRender.com (**a**).

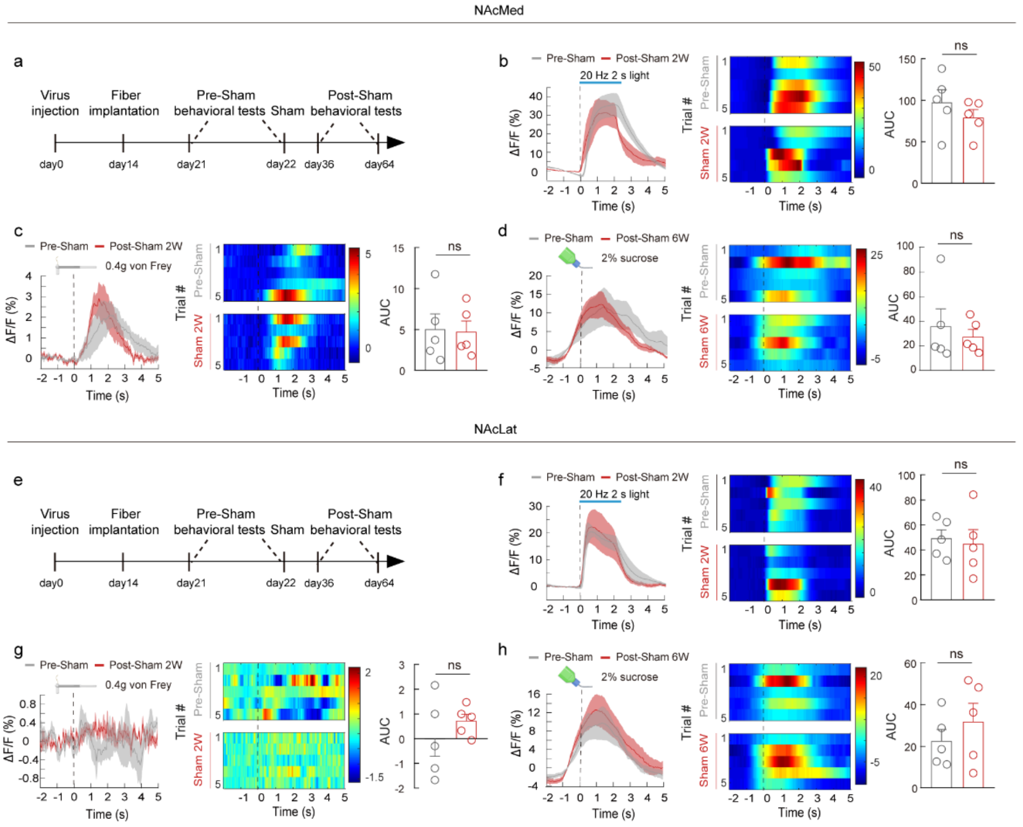


**Supplementary Fig 14. DA2m signals evoked by optogenetic activation of VGlut3$^{DRN\rightarrow VTA}$ terminals in sham mice.**

**a**, **e**, Schematic of the experimental design. **b-d**, **f-h**, Averaged responses (left), heatmaps (middle), and AUC during 0-5 s (right) showing DA2m signals evoked by optogenetic activation of VGlut3$^{DRN\rightarrow VTA}$ terminals (**b**: $P = 0.1252$, **f**: $P = 0.8037$) and 0.4g von Frey stimulation (**c**: $P = 0.8496$, **g**: $P = 0.4663$) in pre- and post-Sham 2W mice, sucrose licking in pre- and post-Sham 6W mice (**d**: $P = 0.5096$, **h**: $P = 0.3273$). NAcMed group, n = 5; NAcLat group, n = 5. Significance was assessed by two-tailed paired Student's *t*-test in (**b-d**, **f-h**). All data are presented as the mean ± s.e.m. Not significant (ns). Details of the statistical analyses are presented in Supplementary Data 1. Created with BioRender.com (**c**, **d**, **g**, **h**).

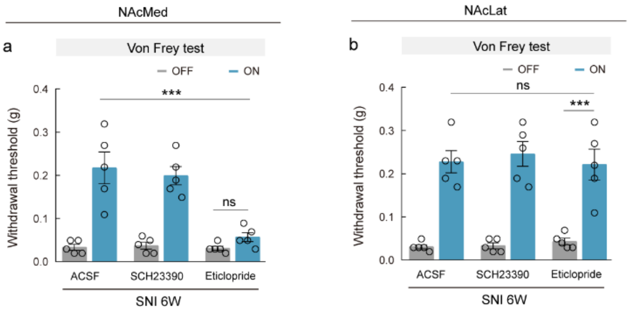


**Supplementary Fig 15. D2 receptors within NAcMed contribute to pain relief through VGluT3$^{DRN}$→DA$^{VTA}$ circuit in post-SNI 6W mice.**

**a**, **b**, Effects of optogenetic activation of VGluT3$^{DRN→VTA}$ terminals on punctate mechanical hypersensitivity with drug infusion into the NAcMed (**a**: ACSF&ON vs Eticlopride&ON $P < 0.0001$; Eticlopride&ON vs Eticlopride&OFF $P > 0.9999$) or NAcLat (**b**: ACSF&ON vs Eticlopride&ON $P > 0.9999$; Eticlopride&ON vs Eticlopride&OFF $P < 0.0001$) in post-SNI 6W mice. NAcMed group, n = 5; NAcLat group, n = 5. Significance was assessed by two-way ANOVA followed by Bonferroni's multiple comparisons test. All data are presented as the mean ± s.e.m. ***$P < 0.001$, not significant (ns). Details of the statistical analyses are presented in Supplementary Data 1.

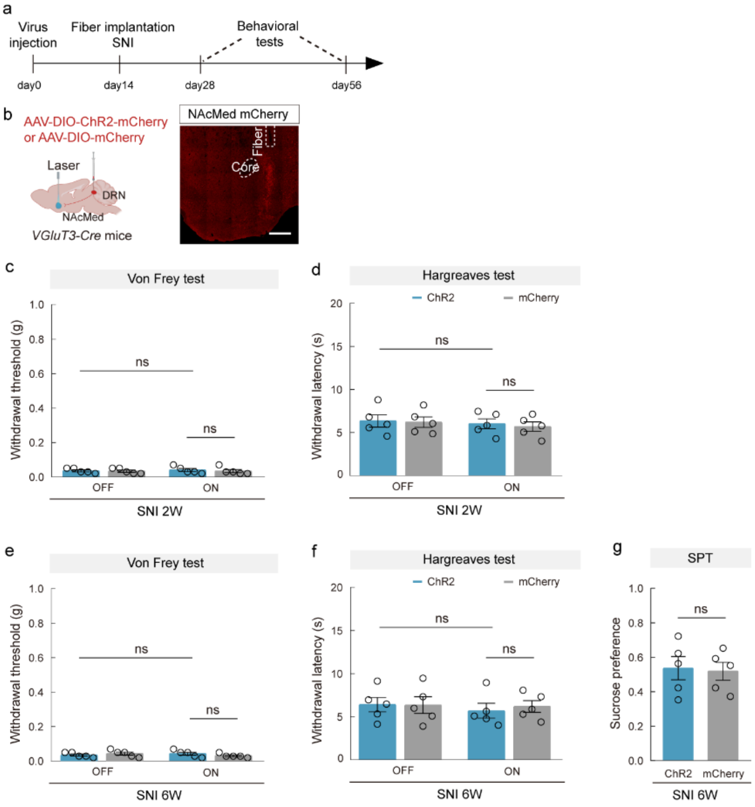


**Supplementary Fig 16. Effects of activation of VGluT3$^{DRN}$ neural terminals within the NAcMed on chronic pain hypersensitivity and CAB.**

**a,** Schematic of the experimental design. **b**, Schematic of DRN injection of AAV-DIO-ChR2-mCherry/AAV-DIO-mCherry and representative images showing NAcMed optical fiber implantation in *VGluT3*-Cre mice. Scale bars, 500 μm. **c-f**, Mechanical paw withdrawal threshold (**c**: ChR2&ON vs ChR2&OFF $P > 0.9999$; ChR2&ON vs mCherry&ON $P > 0.9999$, **e**: ChR2&ON vs ChR2&OFF $P > 0.9999$; ChR2&ON vs mCherry&ON $P > 0.9999$) and thermal paw withdrawal latency (**d**: ChR2&ON vs ChR2&OFF $P > 0.9999$; ChR2&ON vs mCherry&ON $P > 0.9999$, **f**: ChR2&ON vs ChR2&OFF $P > 0.9999$; ChR2&ON vs mCherry&ON $P > 0.9999$) of ChR2-mCherry-expressing and mCherry-expressing SNI mice with (on) or without (off) optogenetic stimulation. **g**, Preference for sucrose in the SPT, $P = 0.8377$. n = 5 mice per group in panels (**c-g**). Significance was assessed by two-way ANOVA followed by Bonferroni's multiple comparisons test in (**c-f**) and two-tailed unpaired Student's *t*-test in (**g**). All data are presented as the mean ± s.e.m. Not significant (ns).

Details of the statistical analyses are presented in Supplementary Data 1. Created with BioRender.com (**b**).

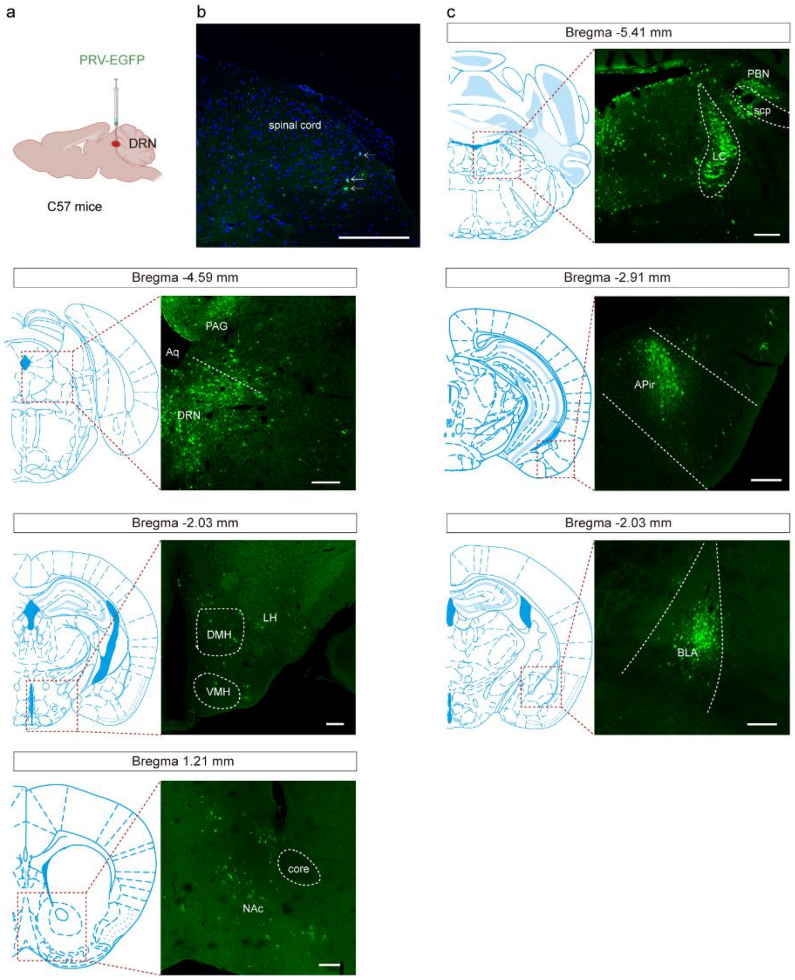


**Supplementary Fig 17. Retrograde and trans-synaptic tracing of DRN-upstream neurons with PRV-EGFP injection.**

**a**, Schematic of the retrograde and trans-synaptic tracing strategy using PRV-EGFP in *C57* mice. **b**, EGFP-expressing neurons detected in the spinal dorsal horn. Scale bars, 200 μm. **c**, Sample images showing EGFP-expressing cells (green) that make trans-synaptic inputs onto DRN neurons. Scale bars, 200 μm. PBN, parabrachial nucleus; PAG, periaqueductal gray; LC, locus coeruleus; DRN, dorsal raphe nucleus; APir, amygdalopiriform transition area; LH, lateral hypothalamus; DMH, dorsomedial hypothalamus; VMH, ventromedial hypothalamus; BLA, basolateral amygdala; NAc, nucleus accumbens. Created with BioRender.com (**a**).

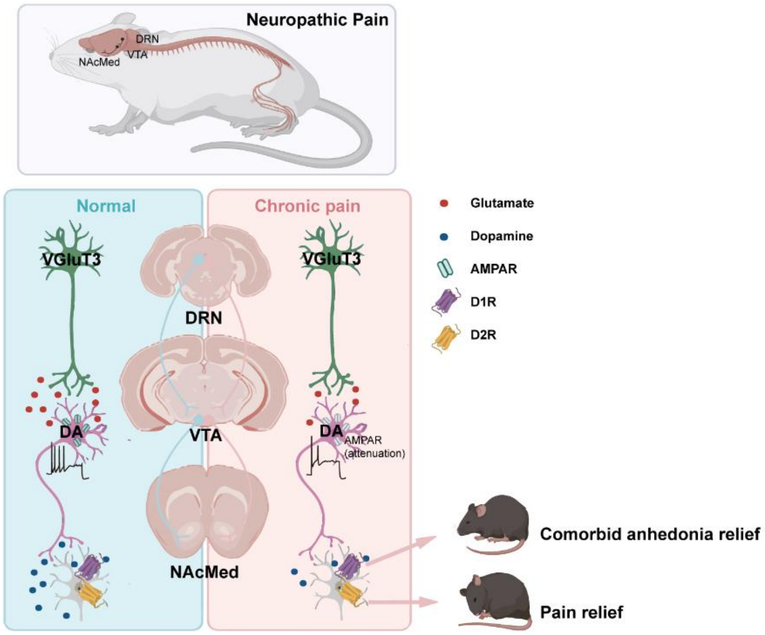


**Supplementary Fig 18. Schematic summary of maladaptive changes of VGluT3$^{\mathbf{DRN}}$→DA$^{\mathbf{VTA}}$→D1/D2$^{\mathbf{NAcMed}}$ circuit during neuropathic pain.**

In the chronic pain state, the VGluT3$^{DRN}$→DA$^{VTA}$ circuit undergoes decreased presynaptic glutamate release and undermined postsynaptic response to glutamate, leading to hypoexcitability of DA$^{VTA}$ neurons and thus reducing dopamine release into the NAcMed. Gain-of-function of VGluT3$^{DRN}$→DA$^{VTA}$ circuit efficiently relieves neuropathic pain and comorbid anhedonia-like behavior via D2R and D1R in the NAcMed, respectively. Created with BioRender.com.

291
