# Supplementary material for: A glutamatergic DRN–VTA pathway modulates neuropathic pain and comorbid anhedonia-like behavior in mice
Source: Nat Commun. 2023 Aug 23;14:5124. doi: 10.1038/s41467-023-40860-3 (PMC10447530; doi:10.1038/s41467-023-40860-3)
Supplement: Supplementary file 3 — Description of Additional Supplementary Files [file 41467_2023_40860_MOESM3_ESM.pdf]

Description of Additional Supplementary Files

File Name: Supplementary Data 1
Description: Details of the statistical analyses.
