# Supplementary material for: A glutamatergic DRN–VTA pathway modulates neuropathic pain and comorbid anhedonia-like behavior in mice
Source: Nat Commun. 2023 Aug 23;14:5124. doi: 10.1038/s41467-023-40860-3 (PMC10447530; doi:10.1038/s41467-023-40860-3)
Supplement: Supplementary file 5 — Reporting Summary [file 41467_2023_40860_MOESM5_ESM.pdf]

nature portfolio

Corresponding author(s): Yan Zhang, Xin-Feng Liu, Zhi Zhang

Last updated by author(s): Jun 28, 2023

Reporting Summary

Nature Portfolio wishes to improve the reproducibility of the work that we publish. This form provides structure for consistency and transparency in reporting. For further information on Nature Portfolio policies, see our Editorial Policies and the Editorial Policy Checklist.

Statistics

For all statistical analyses, confirm that the following items are present in the figure legend, table legend, main text, or Methods section.

n/a	Confirmed	
☐	☒	The exact sample size (*n*) for each experimental group/condition, given as a discrete number and unit of measurement
☐	☒	A statement on whether measurements were taken from distinct samples or whether the same sample was measured repeatedly
☐	☒	The statistical test(s) used AND whether they are one- or two-sided<br>*Only common tests should be described solely by name; describe more complex techniques in the Methods section.*
☒	☐	A description of all covariates tested
☐	☒	A description of any assumptions or corrections, such as tests of normality and adjustment for multiple comparisons
☐	☒	A full description of the statistical parameters including central tendency (e.g. means) or other basic estimates (e.g. regression coefficient) AND variation (e.g. standard deviation) or associated estimates of uncertainty (e.g. confidence intervals)
☐	☒	For null hypothesis testing, the test statistic (e.g. *F*, *t*, *r*) with confidence intervals, effect sizes, degrees of freedom and *P* value noted<br>*Give P values as exact values whenever suitable.*
☒	☐	For Bayesian analysis, information on the choice of priors and Markov chain Monte Carlo settings
☒	☐	For hierarchical and complex designs, identification of the appropriate level for tests and full reporting of outcomes
☒	☐	Estimates of effect sizes (e.g. Cohen's *d*, Pearson's *r*), indicating how they were calculated

Our web collection on statistics for biologists contains articles on many of the points above.

Software and code

Policy information about availability of computer code

Data collection	Fiber photometry:Thinkertech (Nanjing, China)<br>Whole cell recording:patch-clamp electrophysiology data were analyzed with Clampfit pClamp 10.0 software using MultiClamp 700B (Molecular Devices) and Digidata 1550B (Molecular Devices)<br>Von Frey test:von Frey filaments (North Coast Medical)<br>Hargreaves test:Hargreave's Apparatus (Model390; IITC Life Science Inc, Woodland Hills, CA)<br>Fluorescence imaging data:Olympus FV3000 microscope
Data analysis	GraphPad Prism v.8.0.1<br>Olympus FV10-ASW 4.0a Viewer<br>ImageJ 1.48<br>Clampfit software v.10.0<br>MATLAB software R2017a

For manuscripts utilizing custom algorithms or software that are central to the research but not yet described in published literature, software must be made available to editors and reviewers. We strongly encourage code deposition in a community repository (e.g. GitHub). See the Nature Portfolio guidelines for submitting code & software for further information.

April 2023

Data

Policy information about availability of data

All manuscripts must include a data availability statement. This statement should provide the following information, where applicable:

- Accession codes, unique identifiers, or web links for publicly available datasets
- A description of any restrictions on data availability
- For clinical datasets or third party data, please ensure that the statement adheres to our policy

Source data are provided with this paper. There are no restrictions on data availability in the manuscript.

Research involving human participants, their data, or biological material

Policy information about studies with human participants or human data. See also policy information about sex, gender (identity/presentation), and sexual orientation and race, ethnicity and racism.

Reporting on sex and gender	N/A
Reporting on race, ethnicity, or other socially relevant groupings	N/A
Population characteristics	N/A
Recruitment	N/A
Ethics oversight	N/A

Note that full information on the approval of the study protocol must also be provided in the manuscript.

Field-specific reporting

Please select the one below that is the best fit for your research. If you are not sure, read the appropriate sections before making your selection.

☒ Life sciences ☐ Behavioural & social sciences ☐ Ecological, evolutionary & environmental sciences

For a reference copy of the document with all sections, see nature.com/documents/nr-reporting-summary-flat.pdf

Life sciences study design

All studies must disclose on these points even when the disclosure is negative.

Sample size	No sample size calculation was performed. Our sample sizes are similar to those reported in previous publications (PMID: 31451801; PMID: 33686297;PMID: 34663957)
Data exclusions	Mice that were incorrect for viral injection or optic fiber placement were excluded from this study.
Replication	All attempts at replication were successful. Behavioral experiments were replicated multiples times with independent mice, and data were analyzed by two different experimenters blind to the group allocation of the samples. Numbers of replicates (n) are indicated in the figure legends.
Randomization	All animal were randomly assigned to different groups.
Blinding	All investigators were blinded to group allocation during data collection and analysis.

Reporting for specific materials, systems and methods

We require information from authors about some types of materials, experimental systems and methods used in many studies. Here, indicate whether each material, system or method listed is relevant to your study. If you are not sure if a list item applies to your research, read the appropriate section before selecting a response.

Materials & experimental systems

n/a	Involved in the study
☐	☒ Antibodies
☒	☐ Eukaryotic cell lines
☒	☐ Palaeontology and archaeology
☐	☒ Animals and other organisms
☒	☐ Clinical data
☒	☐ Dual use research of concern
☒	☐ Plants

Methods

n/a	Involved in the study
☒	☐ ChIP-seq
☒	☐ Flow cytometry
☒	☐ MRI-based neuroimaging

Antibodies

Antibodies used	Anti-TPH2 (1:500, rabbit, Abcam, Cat# ab111828),<br>Anti-VGluT3 (1:500, rabbit, Synaptic Systems, Cat# 135203)<br>Anti-GABA (1:500, rabbit, Sigma, Cat# A2052)<br>Anti-TH (1:1000, rabbit, Emd millipore, Cat# AB152)<br>Goat anti-rabbit Alexa Fluor 488 (1:500, Jackson ImmunoResearch Labs, Cat# 111-545-003)<br>Goat anti-rabbit Alexa Fluor 594 (1:500, Jackson ImmunoResearch Labs, Cat# 111-585-003)<br>Goat anti-rabbit Alexa Fluor 647 (1:500, Jackson ImmunoResearch Labs, Cat# 111-605-003)
Validation	Anti-TPH:RRID:AB_10862137<br>Anti-Vglut3:RRID:AB_887886<br>Anti-GABA:RRID:AB_477652<br>Anti-TH:RRID:AB_390204<br>Goat anti-rabbit Alexa Fluor 488:RRID:AB_2338046<br>Goat anti-rabbit Alexa Fluor 594:RRID:AB_2338059

Animals and other research organisms

Policy information about studies involving animals; ARRIVE guidelines recommended for reporting animal research, and Sex and Gender in Research

Laboratory animals	In all experiments, male and female mice aged 8-10 weeks were used. C57BL/6J mice were purchased from Beijing Vital River Laboratory Animal Technology, VGluT3-Cre and Ai14 (RCL-tdTOM) mice were purchased from Jackson Laboratories, DAT-Cre and GAD2-Cre mice were gifts from GQ Bi and Z Zhang, respectively. Mice were kept on a 12-h light/dark cycle (lights on at 7am) at a stable temperature of 23 ± 1 °C and a consistent humidity of 50 ± 5%. Food and water were freely available.
Wild animals	No wild animals were used.
Reporting on sex	We included both male and female mice in our study and reported them differently in the figures. We did not find any sex differences in any of our results, therefore we pooled data from different sexes together with equal representation.
Field-collected samples	No sample was collected from the field.
Ethics oversight	All animal protocols were approved by the Animal Care and Use Committee of the University of Science and Technology of China.

Note that full information on the approval of the study protocol must also be provided in the manuscript.
